# Is hyperoxia in early pediatric veno-veno extracorporeal life support associated with mortality?

**DOI:** 10.1051/ject/2024013

**Published:** 2025-06-16

**Authors:** Asaad G. Beshish, Paola Rodriguez-Morales, Rebecca Shamah, Joshua Qian, Kasey Keane-Lerner, Tawanda Zinyandu, Joel Davis, Joshua M. Rosenblum, Heather K. Viamonte

**Affiliations:** 1 Department of Pediatrics, Division of Cardiology, Emory University School of Medicine, Children’s Healthcare of Atlanta 1405 Clifton Rd NE Atlanta GA 30322 USA; 2 Emory University School of Medicine 100 Woodruff Circle Atlanta GA 30322 USA; 3 Children’s Healthcare of Atlanta 1405 Clifton Rd NE Atlanta GA 30322 USA; 4 ECMO and Advanced Technologies, Children’s Healthcare of Atlanta 1405 Clifton Rd NE Atlanta GA 30322 USA; 5 Department of Surgery, Division of Cardiothoracic Surgery, Emory University School of Medicine, Children’s Healthcare of Atlanta 1405 Clifton Rd NE Atlanta GA 30322 USA

**Keywords:** Extracorporeal life support (ECLS), Hyperoxia, Veno-veno extracorporeal life support (VV-ECLS), Mortality, Functional Status Scale (FSS)

## Abstract

*Background*: Data is limited regarding the effects of supraphysiologic blood oxygen tension (hyperoxia) in patients requiring extracorporeal life support (ECLS). We sought to evaluate the association between hyperoxia and outcomes in pediatric patients requiring veno-venous (VV) ECLS. *Methods*: Retrospective single-center study at an academic children’s hospital, Children’s Healthcare of Atlanta, Emory University School of Medicine that included all patients 0–18 years who required VV-ECLS between 01/2014 and 12/2019. *Results*: During the study period 110 VV-ECLS runs occurred in 110 patients. Using a receiver operating characteristic curve, a mean partial pressures of oxygen (PaO_2_) of 122 mmHg in the first 48 hours of ECLS was determined to have the optimal discriminatory ability with regard to mortality (sensitivity 41% and specificity 86%). Of the VV-ECLS runs, 68 (61.8%) had PaO_2_ > 122 mmHg (hyperoxia group). The hyperoxia group tended to be older (105.4 vs. 1.6 months, *p* = 0.001), had higher rates of hemorrhagic complications (69.6% vs. 25.3%, *p* = 0.0001), and higher mortality rate (57.9% vs. 19.5%, *p* = 0.002). Adjusting for confounders, the hyperoxia group had higher odds of mortality (OR: 7.97, 95% CI: 1.72–36.86, *p* = 0.0079). *Conclusion*: Children exposed to hyperoxia during the first 48 h of VV-ECLS were 8 times more likely to die than those who were not after adjusting for confounders (age group, body surface area, and indication for ECLS). Multicenter and prospective evaluation of this modifiable risk factor is imperative to improving the care of this high-risk cohort.

## Introduction

Extracorporeal life support (ECLS) is commonly used to support patients with reversible cardiopulmonary failure refractory to conventional medical treatment. While typically deployed as a bridge-to-recovery, ECLS can also be utilized as a bridge-to-bridge, bridge-to-transplantation, or bridge-to-decision [1, 2]. Since the introduction of ECLS in the mid-1970s, a steady increase in the number and complexity of patients supported has occurred. The most recent ELSO registry report in 2022 demonstrated almost 35,000 neonatal and pediatric patients supported with ECLS with an overall survival rate of about 50% [3].

During veno-veno (VV-) ECLS, deoxygenated blood is removed from the venous side of the circulation, oxygenated, and pumped back to the venous side via the same vein (double lumen cannula) or through a different vein (single lumen cannula). These circuits utilize a highly efficient oxygenator resulting in high partial pressures of oxygen (PaO_2_) that can exceed 400–500 mmHg. Exposure to these supranormal levels of oxygen is termed hyperoxia. Hyperoxia has been well studied in various clinical scenarios in both adults and children where it has been associated with increased morbidity and mortality [2, 4–14]. Although the negative effects of hyperoxia and its association with adverse outcomes are known, the level at which PaO_2_ becomes deleterious may differ depending on the clinical situation. Potential influencing factors include the duration of exposure, the patient’s age, the underlying baseline physiology of the patient, and the overall pathophysiology of the disease process [4, 5, 9, 13–17].

Given the lack of a clear definition of hyperoxia from prior reports, we aimed to evaluate a high-risk patient population who required VV-ECLS for respiratory failure in a high-volume ECLS center. We intended to determine the ranges of PaO_2_ exposure and the potential association between exposure to hyperoxia and poor outcomes. Our primary aim was to determine if hyperoxia while on VV-ECLS was associated with increased mortality using a derived cut-point within our cohort. Our secondary aim was to determine if hyperoxia during VV-ECLS is associated with greater odds of morbidity using the Functional Status Scale (FSS), and the development of complications while on ECLS, including acute kidney injury (AKI).

## Materials and methods

This is a single-center retrospective cohort study that included all patients who required VV-ECLS between January 1st, 2014, and December 31st, 2019, at Children’s Healthcare of Atlanta (CHOA), a free-standing, university-affiliated quaternary children’s hospital. An internal ECLS database was queried, and eligible patient encounters were identified. The study was approved by the CHOA Institutional Review Board (IRB# 00001239, approval date: 10/11/2022). Informed consent was waived.

### Data and definitions

All consecutive patients who required VV-ECLS support in index hospitalization were included. Demographic features, clinical characteristics, and ECLS variables were collected. All arterial blood gases were obtained from the patient arterial line during the first 48 h while on ECLS. The primary outcome was defined as all-cause ECLS mortality. The secondary outcome variables included FSS, AKI (Stage II or Stage III, as defined by the KDIGO criteria) [18], and major complications. Major complications were defined as the presence of either cardiovascular, renal, or mechanical complications.

### Functional Status Scale (FSS)

The FSS consists of six main domains: mental status, sensory, communications, motor function, feeding, and respiratory. Functional status for each domain was categorized from a normal score of 1 to very severe dysfunction with a score of 5, giving total FSS scores ranging from 6 to 30 as previously described [19]. Functional status scoring for this study involved retrospectively scoring baseline status (i.e., on admission) and again at hospital discharge by examining the appropriate documentation. FSS score determination was blinded from hyperoxia status. Newborns who had never achieved a stable baseline function were assigned an FSS score of 6. This was operationalized by assigning a baseline FSS score of 6 to all admissions for infants 0–2 days old and transfers from another facility for infants 3–6 days old as previously reported [20–23]. New morbidity was defined as an increase in the total score of ≥3 points, and unfavorable functional outcome was defined as an increase of ≥5 [24].

### Clinical management

All circuits were blood primed before the start of ECLS with packed red blood cells, 25% albumin, sodium-bicarbonate, calcium-gluconate, and heparin for patients <40 kg. It is common practice for ABGs to be obtained at the discretion of the clinical team, most typically 30 min after initial ECLS-cannulation, and then hourly for the first 3 h. Subsequently, they are typically obtained every 3–6 h and 30 min after an adjustment in ECLS support. Target gas exchange parameters are not dictated by protocol at our center. Goal PaO_2_ ranges have no established normal and the variation we describe is derived from measurements occurring during clinical care. Goal PaCO_2_ was 35–45 mmHg, and goal pH was 7.35–7.45. Once patients are placed on ECLS, the ventilator is placed on “rest settings” of the following: ventilator mode pressure control, peak inspiratory pressure 20 cm H_2_O, peak end-expiratory pressure 10 cm H_2_O, respiratory rate 20/min, inspiratory time 1 s, and FiO_2_ 30%.

### Statistical analysis

Statistical analysis was conducted using SAS version 9.0 software, with a significance level set at *p* < 0.05. The diagnostic utility of mean PaO_2_ in predicting mortality was evaluated using Youden’s index (*J* = sensitivity + specificity − 1) and receiver operating characteristic (ROC) curves. The study population was stratified into hyperoxia and non-hyperoxia groups based on the optimal cut-off value for mean PaO_2_, determined by maximizing the *J* value. Fisher’s exact test was employed for comparing categorical variables, while Student’s *t*-test and the nonparametric Wilcoxon rank-sum test were used for continuous variables, as appropriate. Additionally, a scatterplot was generated to examine the relationship between mean PaO_2_, duration of ECLS run, and survival, with Spearman’s correlation coefficient reported. To assess the impact of hyperoxia on mortality and AKI, univariable and multivariable logistic regression analyses were performed, adjusting for BSA, age group, and indication for ECLS in the multivariable analysis that were determined a priori. The results are presented as odds ratios (OR) with corresponding 95% confidence intervals (CI).

## Results

During the study period, 110 VV-ECLS runs. The median age was 4.9 months (IQR: 0.1, 105.4), and the weight was 5.4 kg (IQR: 3.4, 35.0) with an almost even distribution of males and females. The majority of patients were neonates (57.3%). The median time from admission to cannulation was 39.0 h (IQR: 3.0, 116.0) with a median run duration of 140.5 h (IQR: 98.0, 287.0). Overall mortality rate was 26.4% (Table 1). Supplemental Table 1 shows the relationship of PaO_2_ to mortality and other outcomes. We describe the mean, median and range as well as number of samples for outcomes such as mortality, ECLS complications and Stage II or III AKI in Supplemental Table 1.

**Table 1:** Patient demographics and clinical characteristics of entire VV-ECLS cohort stratified by median PaO_2_ levels in the first 48 h in to non-hyperoxia group (PaO_2_ ≤ 122 mmHg) and hyperoxia group (PaO_2_ > 122 mmHg).

Variables	Total cohort (*n* = 110)	Non-hyperoxia group (PaO_2_ ≤ 122 mmHg) (*n* = 87)	Hyperoxia group (PaO_2_ > 122 mmHg) (*n* = 23)	*p*-value
Age (months)	4.9 (0.1, 105.4)	1.6 (0.0, 81.7)	105.4 (3.4, 154.4)	0.001
Age group				0.003
Neonatal	63 (57.3%)	56 (64.4%)	7 (30.4%)	
Pediatrics	47 (42.7%)	31 (35.6%)	16 (69.6%)	
Weight (kg)	5.4 (3.4, 35.0)	4.3 (3.2, 22.0)	28.3 (5.0, 81.2)	0.002
Height (cm)	69.0 (51.0, 139.0)	56.0 (51.0, 127.0)	128.0 (73.4, 170.0)	0.005
BSA (m^2^)	0.4 (0.2, 1.2)	0.3 (0.2, 1.1)	1.4 (0.4, 2.0)	0.005
Race				0.230
Black	57 (51.8%)	41 (47.1%)	16 (69.6%)	
White	38 (34.5%)	32 (36.8%)	6 (26.1%)	
Hispanic	12 (10.9%)	11 (12.6%)	1 (4.3%)	
Other	3 (2.7%)	3 (3.4%)	0 (0.0%)	
Sex				0.194
Female	58 (53.2%)	43 (50.0%)	15 (65.2%)	
Male	51 (46.8%)	43 (50.0%)	8 (34.8%)	
ECLS indication				0.001
Cardiac	8 (7.3%)	4 (4.6%)	4 (17.4%)	
ECPR	8 (7.3%)	3 (3.4%)	5 (21.7%)	
Pulmonary	94 (85.5%)	80 (92.0%)	14 (60.9%)	
Time from admission to ECLS Initiation	39.0 (3.0, 116.0)	24.0 (2.5, 102.0)	40.0 (3.0, 116.0)	0.560
Initial ECLS flow (L/min)	0.5 (0.4, 2.8)	0.4 (0.4, 1.8)	2.9 (0.7, 4.5)	0.001
Mand PaO_2_ (SD)	97.7 (46.64)	79.2 (23.4)	167.5 (46.8)	<0.001
Median PaO_2_ (mmHg)	86.6 (62.9, 114.6)	77.4 (59.9, 100.5)	159.9 (129.7, 202.2)	<0.001
PaO_2_ range (mmHg)	37.2–277.8	37.2–120.8	122.5–277.8	<0.001
Number of PaO_2_ samples per patient				
Mean (SD)	13.3 (4.21)	12.5 (3.97)	16.2 (3.87)	<0.001
Median (IQR)	12.0	12.0	16.0	<0.001
Duration of ECLS run (hours)	140.5 (98.0, 287.0)	146.0 (98.0, 289.0)	136.0 (89.0, 205.0)	0.549
ECLS complications				
Cardiovascular	40 (40.8%)	28 (37.3%)	12 (52.2%)	0.203
Hemorrhagic	35 (35.7%)	19 (25.3%)	16 (69.6%)	0.0001
Mechanical	57 (58.2%)	43 (57.3%)	14 (60.9%)	0.764
Renal	67 (68.4%)	48 (64.0%)	19 (82.6%)	0.093
Neurologic	14 (14.3%)	11 (14.7%)	3 (13.0%)	0.846
Metabolic	20 (20.4%)	15 (20.0%)	5 (21.7%)	0.856
Infection	3 (3.1%)	3 (4.0%)	0 (0.0%)	0.330
Reason for coming off				
Died or poor prognosis	21 (19.1%)	11 (12.6%)	10 (43.5%)	0.001
ECLS complication	6 (5.5%)	6 (6.9%)	0 (0.0%)	
Expected recovery	80 (72.7%)	69 (79.3%)	11 (47.8%)	
VAD	3 (2.7%)	1 (1.1%)	2 (8.7%)	
AKI stage II/III	61 (64.9%)	50 (66.7%)	11 (57.9%)	0.474
Mortality	29 (26.4%)	17 (19.5%)	12 (52.2%)	0.002

Results depicted in *n* (%) and Median (interquartile range/IQR). ECLS: Extracorporeal Life Support; VV: Veno-veno; VAD: Ventricular Assist Device; AKI: Acute Kidney Injury.

### Cut-point analysis

**Figure 1:**
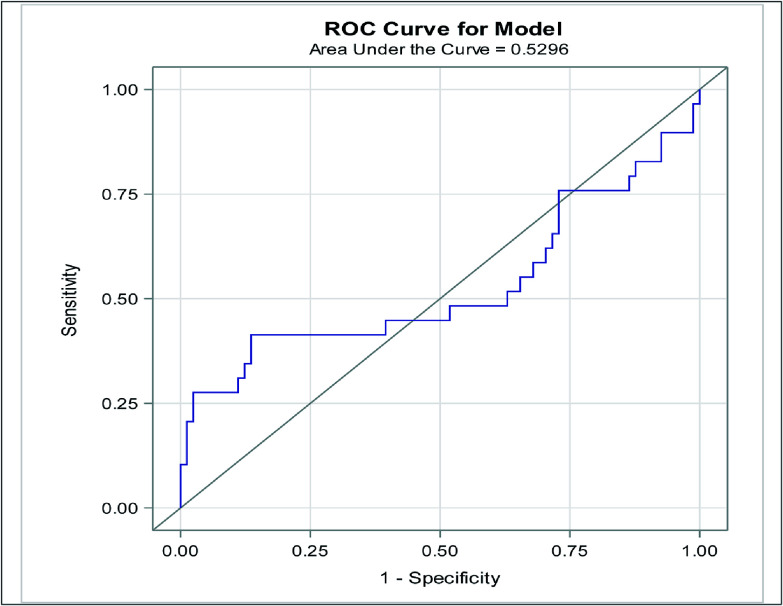
Receiver operating characteristic (ROC) curve identifying the optimal discriminatory cut point for mortality was 122 mmHg (sensitivity 41%, Specificity 86%).

**Figure 2:**
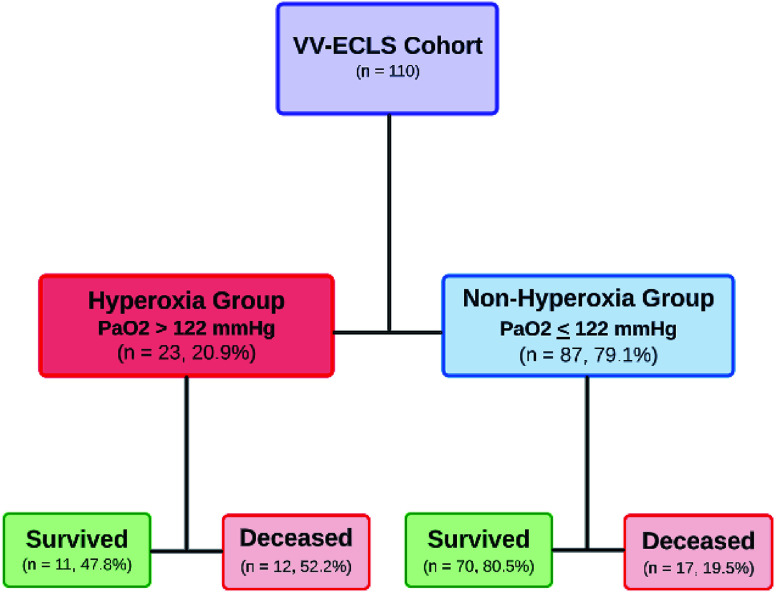
Flow chart of pediatric patients requiring veno-veno extracorporeal life support (VV-ECLS) stratified based on PaO_2_ levels in the first 48-hours while on ECLS.

Using ROC analysis, PaO_2_ > 122 mmHg had the optimal discriminatory ability for operative mortality with a sensitivity of 41%, and specificity of 86% and was defined as hyperoxia for this study population (Figure 1). Area under the curve for PaO_2_ to predict mortality was 0.53 (95% CI: 0.38, 0.68, *p* = 0.962). Patients in the hyperoxia group were older [105.4 months (IQR: 3.4, 154.4) vs. 1.6 (IQR: 0.0, 81.7), *p* = 0.001], more likely to be in the pediatric group vs. neonates 69.6% vs. 30.4%, *p* = 0.003, weighed more 28.3 kg (IQR: 5.0, 81.2) vs. 4.3 (IQR: 3.2, 22.0), *p* = 0.002, and had higher BSA [1.4 kg/m^2^ (IQR: 0.4, 2.0) vs. 0.3 (0.2, 1.1), *p* = 0.005]. Additionally, patients with hyperoxia had a higher rate of hemorrhagic complications while on ECLS 69.6% vs. 25.3%, *p* = 0.0001, and had a higher mortality rate 52.2% vs. 19.5%, *p* = 0.002 (Table 1, Figure 2).

### Outcomes analysis

**Figure 3:**
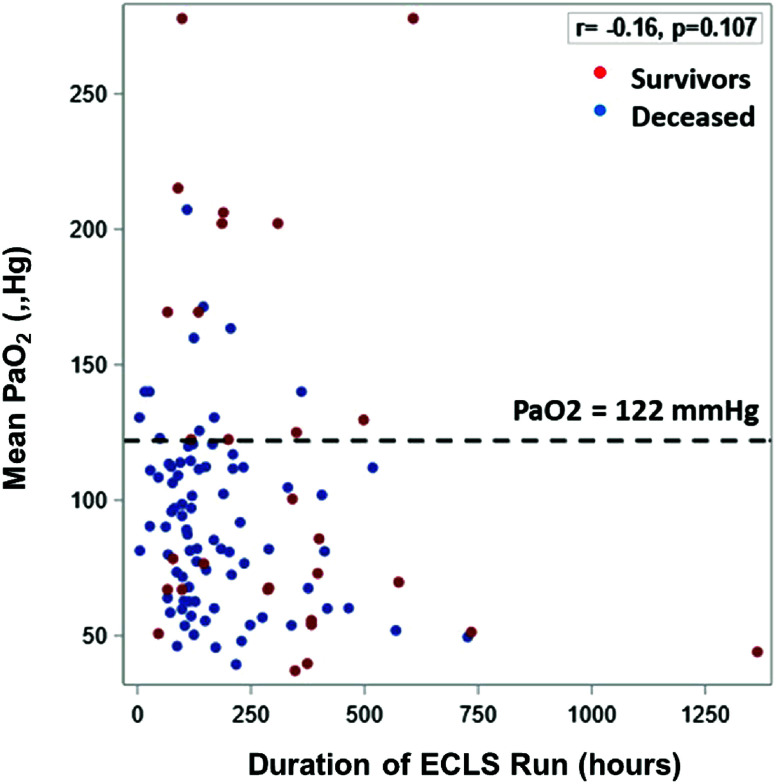
Scatterplot illustrating the relationship of average PaO_2_, VV-ECLS run duration, and mortality in the VV-ECLS cohort.

In univariable analysis, we found that hyperoxia was associated with 4.49 higher odds of mortality (95% CI: 1.70, 11.9, *p* = 0.003) (Table 2). In the multivariable analysis when controlling for age group (neonates vs. pediatrics), BSA, and indication for ECLS, patients in the hyperoxia group had 7.97 higher odds of mortality (95% CI: 1.72, 36.86, *p* = 0.0079). Hyperoxia was not associated with the development of ECLS complications or the development of Stage II or III AKI (Table 2). The association of average PaO_2_ and ECLS duration is graphically demonstrated in Figure 3 [*p* = 0.107, with a correlation coefficient of −0.16 (95% CI: −0.33, 0.03)]. A univariable analysis was conducted in relationship to age group (neonatal vs pediatric), ECLS indication (pulmonary vs cardiac) and body surface area (BSA) with each outcome individually and is shown in Supplemental Table 2.

**Table 2:** Outcomes of patients undergoing VV-ECLS using a univariable and multivariable regression analysis.

Outcomes	PaO_2_ < 122 mmHg (*n* = 87)	PaO_2_ > 122 mmHg (*n* = 23)	OR (95% CI)	*p*-value	aOR^a^ (95% CI)	Adjusted *p*-value
Mortality	29 (33.3%)	12 (52.2%)	4.49 (1.70–11.91)	0.003	7.97 (1.72–36.86)	0.008
Any renal, cardiovascular or mechanical complication	71 (81.6%)	23 (100%)	–	–	–	–
Stage II/III AKI	50 (54.5%)	11 (47.8%)	0.69 (0.45–1.93)	0.476	0.62 (0.18–2.07)	0.431

a Adjusted for age group, BSA, and indication for ECLS.

### Functional Status Scale (FSS) of survivors and development of new morbidity and unfavorable outcomes

Of the 81 survivors, 70 (86.4%) were from the non-hyperoxia group and 11 (13.6%) were from the hyperoxia group. New morbidity (change in total FSS ≥ 3) was demonstrated in 28.6% (20/70) of the non-hyperoxia group, and 18.2% (2/11) of the hyperoxia-group survivors. Unfavorable outcomes (change in total FSS ≥ 5) developed in 7.1% (5/70) of the non-hyperoxia survivors, and 9.1% (1/11) of the hyperoxia survivors (Table 3). We failed to identify an association between designation as “hyperoxia” and new morbidity, or unfavorable outcome (Table 3).

**Table 3:** New morbidity and unfavorable functional outcome for overall survivors who required VV-ECLS stratified by PaO_2_ levels into hyperoxia and non-hyperoxia groups based on functional status scale change from admission to discharge.

ECLS group	Overall cohort of survivors (*n* = 81)	Non-hyperoxia group PaO_2_ ≤ 122 (*n* = 70)	Hyperoxia group PaO_2_ > 122 mmHg (*n* = 11)	*p*-value
New morbidity (change in FSS ≥ 3 points)	22 (27.2%)	20 (28.6%)	2 (18.2%)	0.471
Unfavorable outcome (change in FSS ≥ 5 points)	6 (7.4%)	5 (7.1%)	1 (9.1%)	0.819

FSS Subscale scores range from 1 to 5. Total scores are the sum of subscale scores ranging from 6 to 30. FSS: Functional Status Scale; ECLS: Extracorporeal Life Support.

## Discussion

In this report, we describe a cohort of pediatric patients supported with VV-ECLS with an overall mortality rate of 26.4%. Using a ROC curve, a mean PaO_2_ of 122 mmHg in the first 48 hours of VV-ECLS was determined to have the optimal discrimination for mortality (sensitivity 41% and specificity 86%). Of the 110 VV-ECLS runs, 23 (20.9%) had PaO_2_ > 122 mmHg and were categorized as a hyperoxia group. Patients in the hyperoxia group had 4.5 times higher odds of dying in the unadjusted analysis. This persisted when adjusting for confounders (BSA, ECLS age group, and indication for ECLS) with patients in the hyperoxia group having 7.97 times (95% CI: 1.72, 36.86, *p* = 0.008) higher odds of mortality. While hyperoxia during VV-ECLS may not directly lead to death, we postulate that hyperoxia contributes to comorbidity accumulation that later leads to complications and mortality.

In other critical illness settings, an association between excessive oxygen delivery and poor clinical outcomes has been reported. In patients requiring ECLS for cardiac arrest (CA), hyperoxia (as defined by a mean PaO_2_ > 193 mmHg) was associated with increased 30-day mortality and the need for dialysis [4, 15, 25]. In a large multicenter cohort study of adult patients admitted to the ICU after resuscitation from CA, Kilgannon et al. showed an association between hyperoxia and risk of in-hospital death consistent with a dose-dependent relationship [15]. In a prospective disease-specific CA database, Elmer and colleagues found exposure to severe hyperoxia was independently associated with inpatient mortality [25]. Several reports of neonates with asphyxia have demonstrated an association between hyperoxia and an increased risk of brain injury and mortality [4, 26, 27]. Conversely, Raman et al. in a single-center study and systematic review of a heterogeneous cohort of critically ill patients did not demonstrate an association between hyperoxia at the time of admission and mortality [28]. These reports support earlier findings that hyperoxia is likely associated with worse outcomes, but which populations are at risk remains unclear, and the impact of other clinical variables that may affect oxygenation directly or indirectly. Some of these factors are patient hemoglobin levels, ventilator settings including FiO_2_, the health, and age of the oxygenator in the ECLS circuit, ECLS flows, recirculation, and if the patient is sedated and paralyzed to decrease oxygen consumption. These are all real-life factors that affect the patient at the bedside. It would be extremely useful to control for all of these factors but in reality, the degree of impact of each factor is different for each patient. This supports the importance of this study and future studies to help understand the true impact of oxygen on patient outcomes and the biological systems of the body.

Despite a slew of published data, there is no generally accepted level that defines pathologic hyperoxia, as it may vary by patient population and clinical context [25]. Poor outcomes may occur when PaO_2_ exceeds a certain threshold of antioxidation systems of the body resulting in reactive oxygen species (ROS) production, and activation of inflammatory pathways which result in cellular injury and death [29]. This effect may be more pronounced in neonates, infants, and children due to the immature antioxidant defenses which renders them more susceptible to ROS [4]. Furthermore, the effect of hyperoxia may be more pronounced in patients who are critically ill. When critically ill patients are placed on ECLS they are exposed to a relative hyperoxia state. This exposure to supraphysiologic oxygen may overwhelm the already depleted antioxidant system and result in increased morbidity and mortality. In our study, we show that patients in the hyperoxia group were older (1.6 months vs. 105.4 months, *p* = 0.001, and weighed more (28.3 kg vs. 4.3 kg, *p* = 0.002), and were more likely to be neonates. It appears that in the clinical setting and in particular patients in the neonatal ICU, the providers are more vigilant about limiting oxygen exposure due to the abundance of literature supporting hyperoxia exposure and outcomes in the neonatal population. In the older patient population who are supported on VV-ECLS in our study the most common diagnosis is acute respiratory distress syndrome (ARDS) while in the neonatal population, the most common diagnosis is persistent pulmonary hypertension of the newborn (PPHN), and neonatal respiratory distress syndrome. This shows the importance of a prospective study in both the pediatric and neonatal populations to further identify the appropriate cut-off in each patient population.

Because there is no accepted definition of hyperoxia in pediatric patients supported by VV-ECLS, we used a ROC curve analysis in this specific cohort to determine which PaO_2_ values may be associated with an adverse outcome. This similar strategy was employed by Sznycer-Taub et al. and Beshish et al. in two separate reports. Sznycer-Taub and colleagues evaluated hyperoxia in pediatric cardiac patients (neonates and infants) supported on VA-ECMO and found that a PaO_2_ of 193 mmHg in the first 48 h was determined to have good discriminatory ability with regard to 30-day mortality [4]. Using a similar strategy, Beshish and colleagues showed that a PaO_2_ of 313 mmHg for infants undergoing cardiac surgery utilizing cardiopulmonary bypass was independently associated with 30-day mortality [9]. Although the ECLS modality is slightly different from the prior reports as we describe our experience with VV-ECLS, our cut-off definition of hyperoxia was PaO_2_ of 122 mmHg. The sensitivity of our cut point was 41%, which is slightly low, and we think that this can be better identified with a larger patient population and a homogenous patient population. To do this is extremely challenging for a single center due to the low numbers of VV-ECLS runs in each center. Despite that, we showed the patients in the hyperoxia group had almost eight times higher odds of mortality when adjusting for confounding variables. This is the first report of an association between PaO_2_ level and mortality in patients requiring VV-ECLS and highlights an important modifiable risk factor that clinicians can adjust when taking care of these critically ill patients in hopes of improving overall outcomes, including morbidity and mortality.

## Limitations

Our findings are subject to all limitations inherent to single-center retrospective cohort studies. Although samples to measure PaO_2_ were obtained at dedicated time intervals, it is not possible to discern the effect of time spent in a hyperoxia state as opposed to the effects of acutely high PaO_2_ levels. Additionally, there may be some bias as to which patients are exposed to hyperoxia. For example, we show that patients in the hyperoxia group are older and have a smaller number of neonates. This could be related to the oxygen management strategies in the neonatal population. Despite that, when controlling for age group in the multivariable analysis the association between hyperoxia and mortality persisted. The majority of our cohort had a PaO_2_ level near or above the cut-off of 122 mmHg while on VV-ECLS limiting our ability to study the relationship between lower oxygen tension levels and outcomes. Although we identified a cut point for PaO_2_ of 122 mmHg using an AUC, the sensitivity was 41%. The sensitivity is low, and this is clearly a limitation of our study that we think can be overcome with a larger patient population that is more homogenous. Importantly, many of these limitations can be addressed in a multicenter validation study, which our group is currently pursuing.

## Conclusions

Of the 110 VV-ECLS runs in 107 patients, using an ROC curve the optima paO_2_ associated with mortality was 122 mmHg (sensitivity 41%, specificity 86%). Patients in the hyperoxia group were older, had higher weight and BSA, and had higher mortality rates. Children exposed to hyperoxia during the first 48 hours of VV-ECLS were 8 times more likely to die than those who were not exposed to hyperoxia. Multicenter and prospective evaluation of this modifiable risk factor is imperative to improve the care of this high-risk cohort.

## Data Availability

All available data are incorporated into the article.

## Supplementary material

Supplementary material is available at https://www.ject.edpsciences.org/10.1051/ject/2024013/olm. *Supplemental Table 1*. PaO_2_ by primary outcome. *Supplemental Table 2*. Univariable logistic regression.
