# Supplementary material for: Is hyperoxia in early pediatric veno-veno extracorporeal life support associated with mortality?
Source: J Extra Corpor Technol. 2025 Jun 16;57(2):59–65. doi: 10.1051/ject/2024013 (PMC12169720; doi:10.1051/ject/2024013)
Supplement: Supplementary file 1 — Supplemental Table 1. PaO2 by primary outcome. Supplemental Table 2. Univariable logistic regression. [file ject-57-59-s1.pdf]

Supplemental Table 1. PaO2 By Primary Outcome

PaO_2 Value by Mortality

	Mortality		p-value
	No (n = 81)	Yes (n= 29)	
**PaO2**			**0.03**[1]
Mean (SD)	91.8 (32.6)	114.0 (71.2)	
Median (IQR)	89.2 (62.9, 112.2)	118.5 (67.1, 169.5)	
Range	39.5 – 207.3	37.2 – 277.8	
**Number of Samples**			0.134[1]
Mean (SD)	12.9 (4.2)	14.2 (4.1)	
Median (IQR)	12.0 (10.0, 16.0)	13.0 (11.0, 17.0)	

[1]Kruskal – Wallis p-value

PaO_2 Value by Any Complications

	Any Renal, cardiovascular or Mechanical Complications		p-value
	No (n = 16)	Yes (n= 94)	
**PaO2**			0.617[1]
Mean (SD)	86.3 (22.4)	99.6 (49.4)	
Median (IQR)	90.4 (65.9, 104.3)	118.5 (67.1, 169.5)	
Range	46.2 – 119.9	37.2 – 277.8	
**Number of Samples**			0.168[1]
Mean (SD)	11.8 (3.4)	13.5 (4.3)	
Median (IQR)	11.5 (10.0, 13.0)	13.0 (10.0, 16.0)	

[1]Kruskal – Wallis p-value

PaO_2 Values by Stage II or III Acute Kidney Injury

	Stage II or III Acute Kidney Injury		p-value
	No (n = 49)	Yes (n= 61)	
**PaO2**			0.237[1]
Mean (SD)	89.9 (36.1)	112.8 (59.2)	
Median Range	81.5	102.4	
	39.5 – 202.2	48.1 – 277.8	
**Number of Samples**			0.338[1]
Mean (SD)	11.9. (3.5)	13.5 (4.0)	
Median (IQR)	11.5 (10.0, 13.0)	13.0 (10.0, 16.0)	

[1]Kruskal – Wallis p-value

Supplemental Table 2. Univariable Logistic Regression

Univariate Logistic Regression

Covariate	Stage II or III Acute Kidney Injury	
	**Odds Ratio (95% CI)**	**p-value**
**Age Group**		
Neonatal	1.95 (0.83 – 4.61)	0.126
Pediatric (reference)	–	–
**ECLS Indication**		
**Pulmonary**	1.28 (0.41 – 3.99)	0.665
**Cardiac**	–	–
**Body Surface Area** (BSA)	0.83 (0.45 – 1.53)	0.548

Univariate Logistic Regression

Covariate	Any Renal, Cardiovascular or Mechanical complication	
	**Odds Ratio (95% CI)**	**p-value**
**Age Group**		
Neonatal	0.00 (0.00 – 1.23)	0.936
Pediatric (reference)	–	–
**ECLS Indication**		
**Pulmonary**	0.00 (0.00 – 1.00)	0.964
**Cardiac**	–	–
**Body Surface Area** (BSA)	0.78 (0.41 – 1.42)	0.476

Univariate Logistic Regression

Covariate	Mortality	
	**Odds Ratio (95% CI)**	**p-value**
**Age Group**		
Neonatal	0.89 (0.38 – 2.091)	0.790
Pediatric (reference)	–	–
**ECLS Indication**		
**Pulmonary**	0.07 (0.02 – 0.26)	<0.001
**Cardiac**	–	–
**Body Surface Area** (BSA)	1.28 (0.66 – 2.48)	0.468
